# Role of Neuroimmune Crosstalk in Mediating the Anti-inflammatory and Analgesic Effects of Acupuncture on Inflammatory Pain

**DOI:** 10.3389/fnins.2021.695670

**Published:** 2021-08-02

**Authors:** Baomin Dou, Yanan Li, Jie Ma, Zhifang Xu, Wen Fan, Lixin Tian, Zhihan Chen, Ningcen Li, Yinan Gong, Zhongxi Lyu, Yuxin Fang, Yangyang Liu, Yuan Xu, Shenjun Wang, Bo Chen, Yongming Guo, Yi Guo, Xiaowei Lin

**Affiliations:** ^1^Research Center of Experimental Acupuncture Science, Tianjin University of Traditional Chinese Medicine, Tianjin, China; ^2^National Clinical Research Center for Chinese Medicine Acupuncture and Moxibustion, Tianjin, China; ^3^School of Acupuncture & Moxibustion and Tuina, Tianjin University of Traditional Chinese Medicine, Tianjin, China; ^4^Department of Rehabilitation Physical Therapy Course, Faculty of Health Science, Suzuka University of Medical Science, Suzuka, Japan; ^5^School of Traditional Chinese Medicine, Tianjin University of Traditional Chinese Medicine, Tianjin, China

**Keywords:** acupuncture, inflammatory pain, analgesia, neuroimmune crosstalk, anti-inflammation

## Abstract

Inflammatory pain is caused by peripheral tissue injury and inflammation. Inflammation leads to peripheral sensitization, which may further cause central sensitization, resulting in chronic pain and progressive functional disability. Neuroimmune crosstalk plays an essential role in the development and maintenance of inflammatory pain. Studies in recent years have shown that acupuncture can exert anti-inflammatory and analgesic effects by regulating peripheral (i.e., involving local acupoints and inflamed regions) and central neuroimmune interactions. At the local acupoints, acupuncture can activate the TRPV1 and TRPV2 channels of mast cells, thereby promoting degranulation and the release of histamine, adenosine, and other immune mediators, which interact with receptors on nerve endings and initiate neuroimmune regulation. At sites of inflammation, acupuncture enables the recruitment of immune cells, causing the release of opioid peptides, while also exerting direct analgesic effects via nerve endings. Furthermore, acupuncture promotes the balance of immune cells and regulates the release of inflammatory factors, thereby reducing the stimulation of nociceptive receptors in peripheral organs. Acupuncture also alleviates peripheral neurogenic inflammation by inhibiting the release of substance P (SP) and calcitonin gene-related peptide from the dorsal root ganglia. At the central nervous system level, acupuncture inhibits the crosstalk between glial cells and neurons by inhibiting the p38 MAPK, ERK, and JNK signaling pathways and regulating the release of inflammatory mediators. It also reduces the excitability of the pain pathway by reducing the release of excitatory neurotransmitters and promoting the release of inhibitory neurotransmitters from neurons and glial cells. In conclusion, the regulation of neuroimmune crosstalk at the peripheral and central levels mediates the anti-inflammatory and analgesic effects of acupuncture on inflammatory pain in an integrated manner. These findings provide novel insights enabling the clinical application of acupuncture in the treatment of inflammatory diseases.

## Introduction

Acupuncture, a complementary and alternative therapy, is widely used around the world and has been proven to provide substantial pain relief (Qiao et al., 2020). Of the patients receiving acupuncture treatment for pain relief, 41% have diseases involving inflammatory pain (Wang et al., 2018). Currently, the World Health Organization recommends acupuncture for the treatment of 16 inflammatory pain-related diseases, including rheumatoid arthritis (RA), acute gastritis, chronic gastritis, frozen shoulder, and allergic rhinitis (WHO, 2013). Evidence shows that acupuncture can be a low-cost, low-risk therapy with few and minor adverse effects and it can help in the management of diseases involving inflammatory pain and reduce reliance on analgesics, including morphine (Qin et al., 2016; Wu et al., 2018; A-Wang et al., 2020; Zhang et al., 2020a). In patients with knee osteoarthritis (KOA), acupuncture can significantly relieve pain, attenuate the decline in physical function, and improve quality of life (Corbett et al., 2013). Further, acupuncture appears to be more effective and safe than conventional drug therapy in treating the abdominal pain symptoms associated with ulcerative colitis (UC; A-Wang et al., 2020; Cheng et al., 2020).

Tissue damage or infection can induce an inflammatory response and promote the release of inflammatory mediators from immune cells. These inflammatory mediators activate peripheral nociceptors and nociceptive neurons in the dorsal root ganglion (DRG), causing them to release large amounts of substance P (SP) and calcitonin-gene-related peptide (CGRP; Ronchetti et al., 2017). The release of these signaling molecules aggravates the inflammatory response, mediates peripheral sensitization, and transmits pain signals to the spinal dorsal horn (SDH) via the DRG (Pinho-Ribeiro et al., 2017). Centrally, the stimulation of primary afferent neurons causes the release of inflammatory mediators, which in turn activate central neurons and glial cells. These cells interact with each other, enhancing neuronal function and the circuits involved in pain sensation, and also mediating central sensitization (Muley et al., 2016). Therefore, neuroimmune interactions are crucial for the induction and maintenance of inflammatory pain. Previous studies on the mechanism via which acupuncture relieves inflammatory pain have largely focused on analgesic substances, even though the primary cause of inflammatory pain is the inflammatory response. Recent studies have shown that acupuncture can provide both anti-inflammatory and analgesic effects by regulating neuron and immune cell activity in the central and peripheral systems.

In this review, we first summarize the animal models and acupuncture interventions used for mechanistic studies of acupuncture therapy for inflammatory pain. More importantly, we discuss the neuroimmune crosstalk that is likely involved in these mechanisms at three levels: the local acupoint where acupuncture is performed, the sites of inflammation, and the central nervous system (CNS). Accordingly, we aim to provide a basis for future studies on acupuncture and its application in the treatment of inflammatory pain.

## Methods

### Search Strategy

**FIGURE 1:**
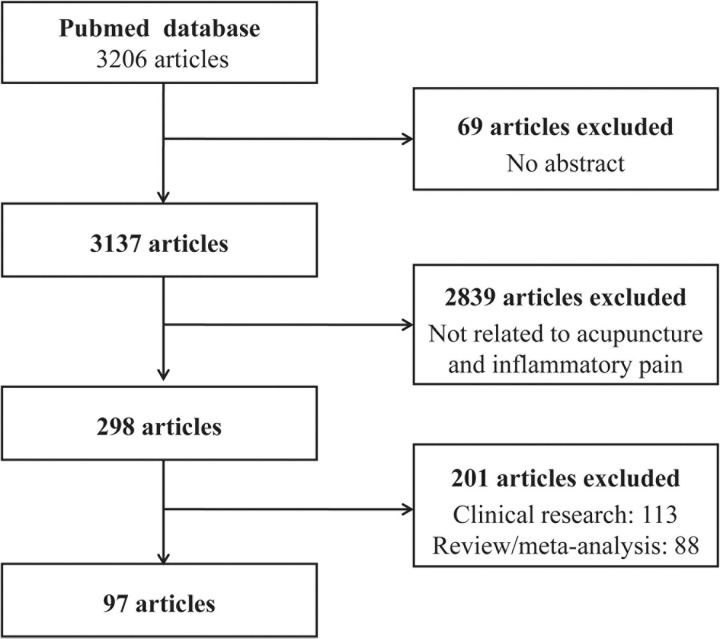
Flow chart of the search strategy and process.

Using the PubMed database, we retrieved studies published between January 2010 and December 2020 using the keywords (“acupuncture” or “electroacupuncture” or “EA” or “manual acupuncture” or “transcutaneous acupoint electrical stimulation” or “TAES”) and (“pain” or “analgesia” or “analgesic”). Only studies in English were included, and 3,206 articles were retrieved in the primary search. Of the identified articles, 69 were excluded due to the absence of an abstract, and the titles and abstracts of the remaining 3,137 articles were screened further to determine whether the studies met the inclusion criteria. At this stage, 2,839 articles were excluded because they were unrelated to acupuncture and inflammatory pain. Of the remaining 298 articles, 97 were basic research articles, 113 were clinical research articles, and 88 were review articles or meta-analyses. The search procedure is depicted in Figure 1.

### Data Extraction

The aim of the literature search was to analyze the mechanism underlying the effect of acupuncture on inflammatory pain. Information from the 97 basic research articles is listed in Table 1 and Supplementary Table 1. First, three authors (BD, YL, and JM) jointly created a database of the studies that examined the mechanism underlying the effects of acupuncture on inflammatory pain. Variables such as the models of inflammatory pain, interventions, and outcome measures were added to this database. The data were extracted by three authors (BD, YL, and JM) and checked by the other authors (ZX, XL, and LT).

**TABLE 1:** Regulation of the anti-inflammatory and analgesic effects of acupuncture.

**Inflammatory pain model**	**Species**	**Intervention methods**	**Acupoints**	**Parameters used for acupuncture**	**Pain measurement**	**Test site** **Site + Mechanism**	**References**
CFA	Mouse	EA	ST36, SP6	2/100 Hz at 2 mA for 20 min	Mechanical allodynia, thermal pain	Paw tissue:IL-10↑, IL-1β↓, NLRP3↓, TNF-α↓ Spleen: Macrophages↓, Neutrophils↓	Yu et al., 2020
CFA	Rat	MA	ST36	Twisting (3 spins per second) for 2 min, followed by a 5-min retention period; total duration, 28 min	Thermal pain	Joints: IL-1β↓, IL-6↓, TNF-α↓ Serum:IL-1α↑, IL-1β↑, IL-6↑, IL-7↑, IL-18↑, TNF-α↑	Xu et al., 2018
POI	Mouse	EA	ST36	10 Hz at 1 mA for 20 min	–	TNF-α↓, IL-6↓, CD45 ^+^ CD11b^ +^↓	Yang et al., 2021
KOA	Rat	MA	ST35, ST36	Twisting (2 spins per second) for 1 min, followed by a 4-min retention; repeated 6 times; total duration, 30 min	Mechanical allodynia, thermal pain	IL-1β↓, TNF-a↓, NGFs↓	Li et al., 2020
CFA	Rat	EA	GB30	100 Hz at 2, 2.5, and 3 mA for 20 min	Mechanical allodynia, thermal pain	Inflamed paw: IFN-γ↑, CXCL10↑	Wang et al., 2014
MIA	Mouse	EA	Ex-LE4, ST35	2 Hz at 1 mA for 30 min	Mechanical allodynia, thermal pain	Menisci: CB2↑, IL-1β↓	Yuan et al., 2018a
CFA	Rat	EA	GB30, GB34	2 Hz at 1 mA for 30 min	Mechanical allodynia, thermal pain	Inflamed skin tissues: NLRP3↓	Gao et al., 2018
IBD	Mouse	EA	BL25	2 Hz at 1 mA for 30 min	Mechanical allodynia	Colon: SP↓, IL-1β↓, A1R↑, A2aR↑, A3R↑, A2bR↓	Hou et al., 2019
CFA	Mouse	EA	ST36	2 Hz at 1 mA for 15 min	Mechanical allodynia, thermal pain	DRG, SCDH: GFAP↓, Iba-1↓, S100B↓, RAGE↓, TRPV1↓	Liao et al., 2017
CFA	Mouse	EA	ST36	2 Hz at 1 mA for 20 min	Mechanical allodynia	DRG: ASIC3↓	Chen et al., 2011
CFA	Rat	EA	ST36, BL60	2, 100, and 2/100 Hz at 0.5, 1.0, and 1.5 mA for 20, 30, and 45 min	Mechanical allodynia, thermal pain	DRG: TRPV1↓, P2 × 3↓	Fang et al., 2018
CFA	Rat	EA	ST36, BL60	100 Hz at 0.5/1.5 mA for 30 min	Mechanical allodynia	DRG, SCDH: P2 × 3↓	Xiang et al., 2019
Inflammatory muscle pain	Mouse	MA	SP6	Insertion depth of about 2–3 mm, rotated slowly for 10 min	Mechanical allodynia, thermal pain	Muscles: IL-10↑, M1 macrophages↓, M2 macrophages↑	da Silva et al., 2015
CFA	Mouse	EA	ST36	2 Hz at 1 mA for 15 min	Mechanical allodynia, thermal pain	DRG, spinal cord, and thalamus: TLR2↓, pPI3K↓, pAkt↓, pmTOR↓, pERK↓, pp38↓, pJNK↓, pCREB↓, pNFκB↓, Nav1.7↓, Nav1.8↓	Hsu et al., 2019
CFA	Mouse	EA	ST36	2 Hz at 1 mA for 20 min	Mechanical allodynia, thermal pain	Cerebellum lobes V, Via, and VII: tTRPV1↓, Nav1.7↓, Nav1.8↓, ASIC3↓, pmTOR↓, pPI3K↓, pAkt↓, Perk↓, RAGE↓, pPKAII α↓, pPKC ε↓, pNF κ B↓, Pcreb↓, S100B↓	Inprasit and Lin, 2020
CFA	Rat	EA	ST36	2 Hz at 1–2 mA for 30 min	Mechanical allodynia, thermal pain	Spinal cord: CX3CL1↓, p38 MAPK↓, IL-1β↓, IL-6↓, TNF-α↓	Li et al., 2019
Neck-incision pain	Rat	EA	LI18, LI4, PC6, ST36, GB34	2/100 Hz at 1 mA for 30 min	Thermal pain	Cervical spinal cord: ATP↑, P2 × 7R/fractalkine/CX3CR1↓	Gao et al., 2017
CFA	Mouse	EA	ST36, SP6	2/15 Hz at 1 mA for 30 min	Mechanical allodynia, thermal pain	Spinal cord: Ica1↑, ICA69↑, ICA69-PICK1↑, PICK1-GluR2↓	Han et al., 2019
CFA	Rat	EA	ST36, SP6	2 Hz at 1 mA for 30 min	Thermal pain	Spinal cord: GluR2↓	Lee et al., 2013
CFA	Rat	EA	GB30	10 Hz at 3 mA for 20 min	Thermal pain	Spinal cord: Use of 5,7-DHT, 5-HT1AR antagonist and 5-HT2CR antagonist affects acupuncture effect	Zhang et al., 2011c
CIOA	Rat	EA	ST36	2/100 Hz at 0.07 mA for 30 min	Mechanical allodynia, thermal pain	Use of 5-HT1, 5-HT2, 5-HT3, muscarinic cholinergic receptor agonist and 5-HT1, 5-HT2, 5-HT3 and muscarinic cholinergic receptors antagonists affects acupuncture effect	Seo et al., 2016
CFA	Rat	EA	GB30	10/100 Hz at 2 mA for 30 min	Thermal pain	RVM: Use of μ, κ-opioid receptor antagonist affects acupuncture effect	Zhang et al., 2011b
MIA	Mouse	EA	Ex-LE4, ST35	2/15/100 Hz at 1/0.1 mA for 30 min	Mechanical allodynia, thermal pain	Midbrain: CB1 receptors↑, 2-AG↑; medulla: 5-HT↑	Yuan et al., 2018b
MIA	Mouse	EA	Ex-LE4, ST35	2 Hz at 1 mA for 30 min	Mechanical allodynia, thermal pain	vlPAG: CB1↑, glutamatergic neurons↑, GABAergic neurons↓	Zhu et al., 2019
MSU	Rat	EA	ST36, BL60	Square wave 0.2 ms at 1–2 mA (each intensity for 15 min; total duration, 30 min)	Ongoing pain score evaluation, mechanical allodynia, thermal pain	Local ankle skin tissue: β-endorphin↑	Chai et al., 2018
CFA	Mouse	EA	ST36, ST37	2 Hz at 1 mA for 30 min	Mechanical allodynia	DRG: Nav sodium currents↓, TRPV1↓	Lu et al., 2016
CFA	Rat	EA	GB30	10 Hz at 3 mA for 20 min	Conditioned place avoidance test	ACC: Use of a selective κ-opioid receptor antagonist affects acupuncture effect	Zhang et al., 2012a
CFA	Rat	EA	ST36, BL60	2/100 Hz at 0.5–1.5 mA for 30 min	Mechanical allodynia	ACC: p-PKMzeta↓, GluR1↓	Du et al., 2017
CFA	Rat	MA	ST36	Twisting (3 spins per second, bi-directionally) for 2 min, followed by a 5-min retention period; total duration, 28 min	Thermal pain	Acupoint: NFκB p-p65↑, NFκB p-p50↑	Zhang et al., 2020c
CFA	Rat	EA	ST36, BL60	0.5–1.5 mA for 30 min	Mechanical allodynia, thermal pain	Acupoint: adenosine↑; DRG: substance P↓, NK1-R↓, TNF-α↓, IL-1β↓, IL-6↓, CD68↓	Zhang et al., 2020d
CFA	Rat	MA	ST36	Twisting (3 spins per second, bi-directionally) for 2 min, followed by a 5-min retention period; total duration, 28 min	Thermal pain	Acupoint: MCP-1↑, CXCL1↑, MIP-1α↑, IL-1β↑, IL-6↑, GM-CSF↓, Macrophages↑	Zhang et al., 2020b
CFA	Mouse	MA	ST36	Two twists every 5 min; total duration, 30 min	Thermal pain	Acupoint: TRPV1↑, TRPV4↑, ASIC3↑	Wu et al., 2014
CFA	Rat	MA	ST36	Lift-thrusting and twisting manipulation for 30 s after 30-s intervals of retention; total duration, 30 min	Thermal pain	Acupoints: Use of histamine, histamine H1 receptor antagonist, mast cell stabilizer, affects acupuncture effect	Huang et al., 2012
CFA	Rat	EA	ST36, BL60	2/100 Hz at 1 mA for the first 15 min and at 2 mA for another 15 min; total duration, 30 min	Thermal pain	DRG, SCDH: TRPV1↓, PKCγ↓, PKCε↓	Liu et al., 2018
Inflammatory arthritis-induced pain	Rat	EA	ST36, SP9, LI4, LR3	2/100 Hz at 2 mA for 20 min	Weight-bearing behavioral tests	Acupoints: Use of opioid, adrenergic, serotonin, and dopamine receptors antagonists affects acupuncture effect	Yoo et al., 2011

*POI, postoperative intestinal; CIOA, CIOA:Collagenase-induced osteoarthritis; MIA, monosodium iodoacetate; MSU, monosodiumurate; GFAP, glial fibrillary acidic protein; Iba1, ionized calcium binding adapter molecule 1; RAGE, receptor for advanced glycation end-products; PI3K, phosphatidylinositol 3-kinase; pAkt, phospho-protein kinasw B; pmTOR, phosphorylated mammalian target of rapamycin; RVM, rostral ventromedial medulla; CXCL1, The chemokine (C-X-C motif) ligand 1; GM-CSF, granulocyte-macrophage colony stimulating factor.*

## Animal Models of Inflammatory Pain Treated Using Acupuncture

### Animal Models of Inflammatory Pain Used in Mechanistic Studies of Acupuncture

Multiple animal models have been used to study the mechanism underlying the effect of acupuncture on inflammatory pain. These include Complete Freund’s adjuvant (CFA)-, carrageenan-, monosodium iodoacetate-, formalin- and collagenase-induced inflammatory pain models, an inflammatory bowel disease (IBD) model, incision-induced pain models, and spontaneous senescence-associated osteoarthritis (OA) models. Of these, the CFA-induced adjuvant arthritis model, which mimics RA pathogenesis, is the primary one used to study the mechanistic effects of acupuncture in the treatment of inflammatory pain owing to the simplicity of model development and the stability of acupuncture efficacy in this model. More importantly, CFA models share similarities with human RA in terms of chronic pain and pathological manifestations such as synovial inflammation, bone destruction, and joint dysfunction and pathological findings such as inflammatory cell infiltration, vascular proliferation, and expansion (Weng et al., 2021), making this model popular in studies on inflammatory pain treated by acupuncture.

### Parameters Related to Acupuncture Interventions in Animal Studies

Of the reviewed animal studies, 70 used ST36 (*Zusanli*) as the site of acupuncture intervention, whereas 18 used GB30 (*Huantiao*) and BL60 (*Kunlun*), 11 used SP6 (*Sanyinjiao*), and 10 used GB34 (*Yanglingquan*). This was consistent with the acupoints typically used for the clinical treatment of OA. Electroacupuncture (EA) was the most frequently used intervention in animal studies, and low-frequency (1, 2, and 10 Hz) or variable-frequency (2/100 Hz) stimulation, a stimulation intensity of 1–2 mA, and a stimulation duration of 30 min were the most commonly used parameters for EA. Further, some studies mentioned that EA inhibits inflammatory pain more effectively at 2–10 Hz than at 100 Hz (Kim et al., 2004; Zhang R. X. et al., 2005). Manual acupuncture (MA) was used in 14 of the included studies. In most MA studies, stimulation was performed 2–3 times per second, and the duration of continuous stimulation was relatively short—generally 1–2 min with an interval of 4–5 min—and the total treatment duration was approximately 30 min. However, few studies compared therapeutic effects between MA and EA, and most of the included studies using EA did not provide detailed descriptions of the wave type and amplitude of electrical stimulation.

## Microenvironment Regulation at the Acupoint After Acupuncture

The initial effects of acupuncture take place at the acupoint, and mechanical stimulation is converted into chemical signals at these sites (Mingfu et al., 2013; Wu et al., 2014). Acupuncture significantly increases extracellular adenosine levels at the ST36 acupoint. In mouse models of inflammatory pain, MA or a local injection of a specific adenosine A1 receptor (A1R) agonist at ST36 significantly inhibits mechanical allodynia and thermal hyperalgesia (Goldman et al., 2010). In CFA-treated rats, EA at ST36 and BL60 reduces the levels of SP, neurokinin-1 receptor (NK-1R), interleukin-6 (IL-6), IL-1β, and tumor necrosis factor (TNF)-α in the DRG by promoting adenosine release and activating the A1Rs of nerve endings at local acupoints. Therefore, it appears that the acupuncture-activated acupoint–A1R pathway contributes to the anti-inflammatory action of acupuncture (Zhang et al., 2020d). Further, in CFA-induced rat models of pain, MA at ST36 can directly induce neural regulation by activating the mechanically sensitive transient receptor potential ion channel vanilloid 1 (TRPV1) channel receptors expressed on nerve terminals and immune cells and promote adenosine triphosphate (ATP) release via calcium wave propagation across nearby nerve endings. The injection of capsaicin, a TRPV1 agonist, can recapitulate the analgesic effect of acupuncture when injected at ST36 (Wu et al., 2014). In CFA models, EA at ST36 can activate mast cells and promote mast cell degranulation by activating TRPV2 and inducing the release of histamine and adenosine, which increases the levels of β-endorphins (β-ENDs) in the cerebrospinal fluid and results in analgesia (Huang et al., 2018). Meanwhile, acupuncture can also produce immune regulation at local acupoints by recruiting and activating immune cells and activating the nuclear factor kappa B (NFκB) pathway, which then induces the release of immune mediators [monocyte chemoattractant protein-1 (MCP-1) and IL-6] that bind to receptors at adjacent nerve endings, thereby transmitting acupuncture signals (Huang et al., 2018; Zhang et al., 2020b).

**FIGURE 2:**
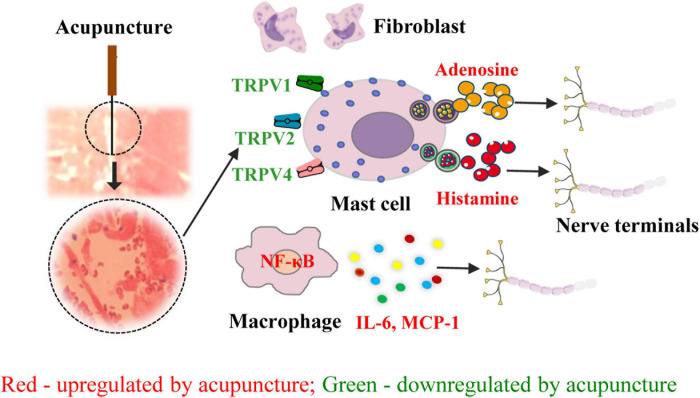
Role of neuroimmune crosstalk at the acupoint in mediating the anti-inflammatory and analgesic effects of acupuncture on inflammatory pain. The names of immune factors are presented in red and green, respectively. The factors in red are up-regulated by acupuncture, while those in green are down-regulated by acupuncture. NF-κB, nuclear factor kappa-B; TRPV1/2/4, transient receptor potential ion channel vanilloid 1/2/4; MCP-1, monocyte chemoattractant protein-1; IL-16, interleukin- 6.

The initiation of the effects of acupuncture involves several factors and is dependent on several substances in the local acupoint microenvironment. As demonstrated by the aforementioned evidence, acupuncture may directly activate nociceptive terminals and immune cells, particularly mast cells, via mechanically sensitive channel receptors. This leads to the release of bioactive chemicals such as ATP and its degradation product, adenosine, which activate nociceptive nerve endings (Figure 2). However, the complicated neuroimmune network in the acupoint microenvironment remains to be elucidated.

## Acupuncture-Induced Inhibition of Peripheral Sensitivity in Inflammatory Pain Models

Inflammation is a hallmark of inflammatory pain. The increased release of inflammatory mediators results in the sensitization of peripheral nociceptors, which is characterized by a lowered activation threshold in these nociceptors (Yu et al., 2020). Activated DRG nociceptor neurons produce and release a large number of neuropeptides, such as SP and CGRP, from their peripheral terminals, which further aggravates local inflammatory responses and peripheral sensitization (Basbaum et al., 2009). Hence, the literature suggests that neuroimmune interactions are crucial in this process. Recently, acupuncture has also been found to alleviate inflammatory pain via neuroimmune crosstalk.

### Regulation of the Immune Cell Polarization Balance

T cells can be classified into several sub-populations, including T helper 1 (Th1) cells, Th2 cells, Th17 cells, and T regulatory (Treg) cells. These cells regulate immune responses by producing specific pro- or anti-inflammatory cytokines (Shaw et al., 2018). For instance, Th1 cells release pro-inflammatory factors such as IL-1, IL-2, interferon-γ (IFN-γ), and TNF-α, while Th2 cells produce IL-10, IL-5, IL-13, IL-4, IL-6, and other anti-inflammatory factors. Pro-inflammatory factors bind to their corresponding receptors and stimulate peripheral nerve endings, while anti-inflammatory factors reduce the excitability of peripheral nerves. A significant imbalance in the Th1/Th2 ratio is observed in RA patients and animal models of RA. In CFA-induced rat models of pain, MA at ST36 induces anti-inflammatory and analgesic effects at inflamed joints. Analyses of cell–cell communication networks have shown that Th1 and Th2 cells act as key mediators in the CFA model and are also the main mediators of MA action (Xu et al., 2018). In the inflamed plantar tissues of mice treated with CFA, EA can inhibit inflammatory responses and pain by reducing the levels of IL-2 and INF-γ produced by Th1 cells and increasing the levels of IL-13, IL-4, and IL-5 produced by Th2 cells. Therefore, the maintenance of the Th1–Th2 balance decreases the sensitivity of peripheral nociceptors and mediates the anti-inflammatory and analgesic effects of EA (Wang et al., 2017).

Treg cells are anti-inflammatory, whereas Th17 cells are pro-inflammatory (Benedetti and Miossec, 2014). Multiple studies have confirmed that in collagen-induced arthritis (CIA) and UC models, EA at ST36 and GB39 (*Xuanzhong*) or RN4 (*Guanyuan*) induces Treg cell proliferation and simultaneously inhibits Th17 differentiation in the spleen, leading to the increased expression of anti-inflammatory factors like Transforming growth factor (TGF-β), IL-10, and IL-2 and the reduced expression of pro-inflammatory factors like IL-6, IL-17A, and IL-17F, ultimately reducing inflammatory hyperalgesia (Jimeno et al., 2012; Zhu et al., 2015; Sun et al., 2017). Similarly, EA at ST36 and SP6 can promote the activation of Treg cells and decrease the expression of the pro-inflammatory mediators IL-1β, TNF-α, and NOD-like receptor family 3 (NLRP3) in the hind paws of CFA-treated mice, reducing the stimulation of peripheral nociceptors in these animals and thereby relieving inflammatory pain (Yu et al., 2020).

Macrophages are highly heterogeneous and plastic immune cells that can polarize into different phenotypes. The polarization of these cells is dependent on different microenvironmental signals, and the polarized macrophages have specific functions in tissue homeostasis or host defense (Murray, 2017). The balance between pro- and anti-inflammatory macrophages also plays an important role in the regulation of inflammatory pain. In CFA-induced rat models of adjuvant arthritis, MA at ST36 can alleviate joint inflammation and pain by increasing the M2/M1 ratio. Further, such stimulation can decrease the levels of the pro-inflammatory cytokines TNF-α, IL-1α, IL-1β, IL-6, IL-7, and IL-18 and increase the levels of the anti-inflammatory cytokines IL-4 and IL-5 (Fuming et al., 2021). In inflammatory muscle pain models, MA at SP6 induces phenotypic changes in muscle macrophages by reducing the number of M1 macrophages and increasing the number of M2 macrophages (major source of IL-10) in the gastrocnemius muscle, thereby reducing thermal and mechanical hyperalgesia and decreasing avoidance behaviors and edema (da Silva et al., 2015). In the OA model, the activation of the NLRP3 inflammasomes leads to the production of IL-1β and TNF-α, resulting in the promotion of synovial inflammation, cartilage degeneration, and chondrocyte apoptosis (Nasi et al., 2017). In OA models, EA at Ex-LE4 (*Neixiyan*) and ST35 (*Dubi*) can significantly inhibit the activation of the NLRP3 inflammasome and the protein expression of matrix metalloproteinase-13, caspase-1, and IL-1β in cartilage tissue, thereby reducing mechanical hyperalgesia, improving the structure of articular cartilage, and reducing cartilage surface fibrillation (B-Wang et al., 2020). Recently studies have shown that EA at ST36 excites the vagus nerve via its dorsal nucleus, activates the α7 nicotinic acetylcholine receptor (α7nAChR)-mediated Janus kinase 2/signal transducer and activator of transcription 3 (JAK2/STAT3) signaling pathway in macrophages, inhibits the expression of the inflammatory factors IL-6 and TNF-α, reduces the local immune responses in the gastrointestinal tract, and promotes the recovery of gastrointestinal motility in mice with postoperative intestinal paralysis (Yang et al., 2021). These pieces of evidence suggest that macrophage polarization and the balance of related cytokines may be the main targets of acupuncture in its regulation of inflammatory pain.

Monocyte- and macrophage-related chemokines are also regulated by acupuncture. The MCP-1/chemokine receptor 2 (CCR2) axis mediates the recruitment of monocytes and macrophages in the early stage of inflammatory pain, causes the secretion of inflammatory factors and nerve growth factors (NGFs), and aggravates OA progression (Raghu et al., 2017). In the KOA model, MA at ST35 and ST36 can inhibit the MCP-1/CCR2 axis, thereby inhibiting the recruitment of monocytes/macrophages and down-regulating IL-1β, TNF-α, and NGFs and alleviating hyperalgesia and cartilage degeneration in the knee joint (Li et al., 2020). In rats treated with CFA, EA at GB30 up-regulates chemokine C-X-C motif chemokine ligand 10 (CXCL10) expression and increases the number of chemokine C-X-C motif receptor 3 (CXCR3)^+^ (receptor of CXCL10) macrophages containing opioids at sites of inflammation (Wang et al., 2014).

Together, this evidence shows that acupuncture can promote the balance of immune cells by regulating immune mediators, thus reducing the inflammatory response at local nociceptors. However, it remains unclear how acupuncture, as a body distal stimulation, inhibits the inflammatory response. Recent studies have reported that acupuncture can activate distinct sympathetic or parasympathetic nerves that project to immune-related organs and modulate systemic inflammation. Future studies of acupuncture treatment for inflammatory pain will be directed toward mapping the somatosensory pathways that drive distinct autonomic pathways.

### Acupuncture-Mediated Promotion of the Release of Analgesic Substances From Immune Cells

#### Opioid Peptides

Electroacupuncture at GB30 can increase the number of opioid-containing macrophages and therefore increase the release of β-ENDs in the inflamed paw tissue of rats treated with CFA (Zhang G. G. et al., 2005). Interestingly, the long-term analgesic effect of EA is antagonized by β-END and enkephalin (ENK) antagonists, but not by dynorphin A (DYN) antagonists, indicating that the β-ENDs released due to acupuncture stimulation act on μ- and δ-opioid receptors and inhibit peripheral inflammatory pain (Wang et al., 2014). In monosodium urate-induced rat models of acute gouty arthritis, variable frequency EA (2/100 Hz) at ST36 and BL60 can up-regulate the expression of β-END in the local ankle tissue, and this β-END can act on μ- and κ-opioid receptors to exert peripheral analgesic effects (Chai et al., 2018). In addition, in carrageenan-induced rat models of pain, EA at ST36 activates μ-, δ-, and κ-opioid receptors, exerting analgesic effects by reducing the excitability of peripheral neurons and inhibiting the release of pro-inflammatory neuropeptides (such as SP) at peripheral nerve endings (Taguchi et al., 2010). These studies show that acupuncture may promote the aggregation of immune cells containing opioid peptides in inflammatory tissues and promote the release of β-END and ENK, which may act on μ-, δ-, and κ-opioid receptors at peripheral sensory endings to exert peripheral analgesic effects.

#### Cannabinoids

The endocannabinoid system is a vital neuromodulation system for pain sensation, and it includes two G protein-coupled receptors: cannabinoid receptor 1 (CB1R) and CB2R. EA at GB30 and GB34 enhances the expression of CB2R in immune cells (keratinocytes, macrophages, and T-lymphocytes) in inflamed skin tissue and reduces pain in CFA-treated rats (Chen et al., 2009; Zhang et al., 2010). In the CFA-treated and KOA pain models, EA can inhibit the release of the pro-inflammatory factors TNF-α, IL-1β, and IL-6 by activating CB2R, thereby suppressing peripheral inflammatory pain (Su et al., 2012; Yuan et al., 2018a; Cristino et al., 2020). EA can also exert anti-inflammatory and analgesic effects by reducing the activation of NLRP3 inflammasomes in skin macrophages via the activation of CB2R, and CB2R knockout can reduce NLRP3 inflammasome activation and weaken the analgesic effect of EA (Gao et al., 2018). In addition, cannabinoids and β-ENDs synergistically mediate the anti-inflammatory and analgesic effects of acupuncture. Interestingly, EA at GB30 and GB34 increases the levels of β-END by activating CB2R in keratinocytes, macrophages, and T lymphocytes in inflamed skin tissue to inhibit inflammatory pain (Su et al., 2011).

#### Adenosine

There are four receptors for adenosine: A1R, A2aR, A2bR, and A3R. Adenosine mainly inhibits inflammatory pain through A1R and A2aR (Andreas et al., 2008; Sawynok, 2016). A2aRs bind to adenosine-activated protein kinase A (PKA), which promotes the production of immunosuppressive cells and increases their infiltration, further inducing an anti-inflammatory response (Lv et al., 2021). EA at BL25 increases the expression of A1R, A2aR, and A3R in the colon of mouse models of IBD. Further, A2bR mediates the acupuncture-induced inhibition of the release of the pro-inflammatory factor IL-1β and reduces visceral pain (Hou et al., 2019). Similarly, EA at ST36 and SP6 can increase A2aR expression and inhibit the release of TNF-α in the ankle joint of CIA mice, thus exerting anti-inflammatory and tissue protection effects (Li et al., 2015). However, the role of A1R in the immune regulation caused by acupuncture has not been explored.

### Neuroimmune Regulation in the Dorsal Root Ganglion by Acupuncture

Peripheral nerve fibers and their cell bodies in the DRG relay inflammatory pain-related afferent input to the spinal cord (Lai et al., 2019). In the DRG, nociceptive ion channels (such as TRPV1) on primary sensory neurons open after peripheral noxious stimulation and activate neurons while releasing active substances such as SP and CGRP. Repeated long-term stimulation causes neurogenic inflammation and enhances neuronal excitability and primary afferent input, causing peripheral sensitization (Yoo et al., 2011).

Transient receptor potential ion channel vanilloid 1 is generally considered to be involved in inflammation and perceived thermal pain. Studies have found that acupuncture can down-regulate the expression and sensitivity of TRPV1 by reducing the release and expression of pro-inflammatory neuropeptides (SP and CGRP), pro-inflammatory cytokines (TNF-α, IL-1β, and IL-6), and NGF and reducing the sensitization of sensory neurons (McDonald et al., 2013). For example, TRPV1 is the main downstream target of Mas-associated G protein-coupled receptor C (MrgprC) activation in the acute inflammatory pain signaling pathway (Huang et al., 2006). EA at bilateral ST36 and BL60 in CFA-treated rats can inhibit the phosphorylation of TRPV1 residues by protein kinase C (PKC) via the down-regulation of MrgprC. This could reduce the sensitivity and openness of TRPV1 channels, thereby reducing internal Na^+^ and K^+^ flow and inhibiting pain transmission (Liu et al., 2018). EA at bilateral ST36 can inhibit the expression of TRPV1 and its downstream signaling molecules in the DRG in CFA-induced animal models of pain, thus down-regulating the activation of p-PKA, p-extracellular signal-regulated kinases (ERK), p-c-Jun-N-terminal kinase (JNK), p38-mitogen-activated protein kinase (MAPK), several transcription factors, p-cAMP response element binding protein (CREB), p-NFκB, and the noxious ion channel Nav1.7 (Liao et al., 2017; Yang et al., 2017). In TRPV1^–/–^ mice, EA treatment does not produce analgesic effects, indicating that TRPV1 is key for EA-mediated analgesia in cases of inflammatory pain (Lu et al., 2016). EA at ST36 can inhibit the expression of acid sensation ion channels 3 (a sensor of acidic environments and mechanical stimuli) and TRPV4 (a sensor of osmotic pressure and mechanical stimuli) in DRG neurons and relieve inflammatory pain in both carrageenan- and CFA-induced models of pain via the inhibition of peripheral sensitization (Chen et al., 2011, 2012).

Substance P is a neuropeptide released by sensory nerve endings and it is the main mediator of neurogenic inflammation. The combination of SP and NK-1R can increase the secretion of pro-inflammatory cytokines and aggravate inflammatory pain (Yang et al., 2013). EA at ST36 can reduce the levels of SP and NK-1R and the expression of IL-6, TNF-α, and IL-1β in the DRG of CFA-treated rats, abating mechanical sensitivity and thermal pain. Interestingly, when the dorsal nerve root is cut or an SP receptor antagonist is injected, the inhibitory effect of EA on SP, NK-1R, and other downstream inflammatory factors is attenuated, which significantly reduces the analgesic effect of EA. Hence, EA may also prevent the transmission of pain signals to the CNS by inhibiting the expression of SP in the DRG (Zhang et al., 2020d).

The purinergic P2 × 3 receptor (P2 × 3R) is a ligand-gated non-selective cation channel that is selectively expressed in primary sensory neurons. When inflammatory pain occurs, ATP is released from damaged tissues or inflammatory cells and binds to P2 × 3R in DRG neurons to activate nociceptors and transmit pain signals (Bernier et al., 2018). EA at ST36 and BL60 can significantly reduce the number of P2 × 3R-positive neurons in the L4-6 DRG and reduce the expression of the P2 × 3R protein in the L6 DRG. Further, P2 × 3R agonists can reduce EA-mediated analgesia in CFA-treated rats (Fang et al., 2018). In CFA-treated rats, both short- and long-term 100-Hz EA at ST36 can reduce the expression of P2 × 3R in the DRG. Moreover, the inhibition or activation of P2 × 3R in the DRG may contribute to or weaken the analgesic effect of EA, respectively (Xiang et al., 2019). In addition, in rat models of IBD, EA at ST37 and ST25 can down-regulate the expression of P2 × 3R in the colon myenteric plexus and reduce sensitivity to visceral pain (Weng et al., 2015).

Tyrosine hydroxylase (TH) is the rate-limiting enzyme in the synthesis of catecholamines, and it is a key component of the peripheral sympathetic nervous system (Dunkley and Dickson, 2019). When the DRG receives pain signals, sympathetic nerve fibers protrude and invade the region around sensory neurons, participating in pain signal transduction. For example, in trinitrobenzene sulfonic acid (TNBS)-induced rat models of colitis, the germination of TH immunoreactive fibers toward DRG sensory neurons contributes to the maintenance and aggravation of peripheral inflammatory pain. EA at ST36 and ST37 can reduce hyperalgesia and inflammatory damage in the distal colon by inhibiting the expression of TH in the L6 DRG. This may be one mechanism via which EA relieves the symptoms of colitis (Wang et al., 2019).

**FIGURE 3:**
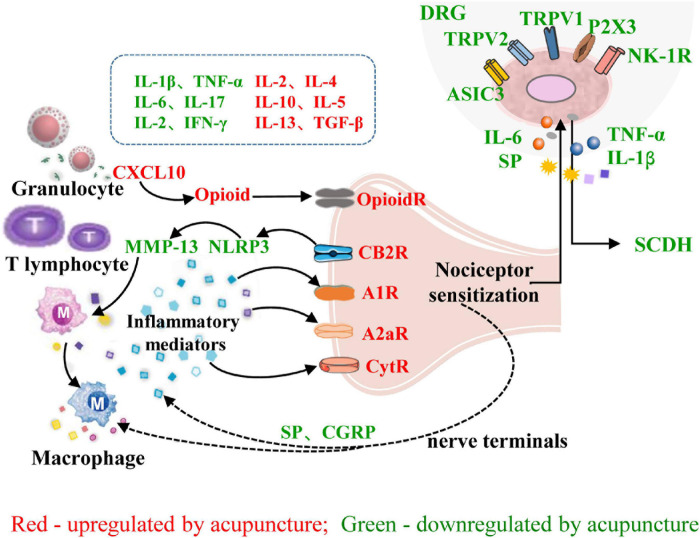
Role of neuroimmune crosstalk at the sites of inflammation in mediating the anti-inflammatory and analgesic effects of acupuncture on inflammatory pain. The names of immune factors are presented in red and green, respectively. The factors in red are up-regulated by acupuncture, while those in green are down-regulated by acupuncture. IL-1β/2/4/5/6/10/13/17, interleukin-1β/2/4/5/6/10/13/17; IFN-γ, interferon-γ; TGF-β, transforming growth factor; CXCL10, chemokine (C-X-C motif) ligand 10; NLRP3, NOD-like receptor family 3; MMP13, matrix metalloproteinase-13; A1R, adenosine 1 receptor; CytR, cytokine receptor; OpioidR, opioid receptor; A2aR, adenosine 2a receptor; SP, substance P; CB2R, cannabinoid receptor 2; P2 × 3, Purinergic P2 × 3 receptor; NK-1R, neurokinin-1 receptor; ASIC3, acid sensation ion channels 3; CGRP, calcitonin-gene-related peptide; TRPV1/2, transient receptor potential ion channel vanilloid 1/2; DRG, dorsal root ganglion; SCDH, spinal cord dorsal horn.

In summary, the interactions between the peripheral nervous system and immune system that underly the effect of acupuncture on inflammatory pain include the following: (1) Acupuncture can induce the migration of immune cells containing analgesic substances to target organs, where they release opioid peptides, cannabinoids, and other analgesic substances to achieve analgesia by blocking peripheral sensitization. (2) Acupuncture regulates the balance of immune cells and reduces the release of pro-inflammatory factors, thereby reducing the stimulation of peripheral nociceptive nerves by inflammatory factors. (3) Acupuncture inhibits the release of SP, CGRP, and other neuropeptides from nociceptive DRG neurons, inhibits TRPV1 and its downstream signaling pathways, reduces peripheral neurogenic inflammation, and inhibits the transmission of pain signals to the CNS (Figure 3).

## Acupuncture-Induced Inhibition of Central Sensitivity in Inflammatory Pain Models

### Regulation of Neuron–Glia Interactions by Acupuncture

Emerging clinical and preclinical studies have indicated that neuron–glia interactions in the spinal cord are involved in the pathogenesis of chronic pain (Gwak et al., 2017; Haight et al., 2019). Inflammatory pain signals are transmitted via peripheral afferent nerves. When a nerve impulse arrives, neurotransmitters such as glutamate, ATP, SP, and CGRP are produced and released from primary DRG neurons, and information is transmitted to the spinal cord. These neurotransmitters act on the receptors present on glial cells and neurons, modulating glial and neuronal activity (Wei et al., 2008; Roberts et al., 2009). Glial cells (microglia and astrocytes) respond to increased input from peripheral nerves by changing their morphology, increasing in number, and releasing pronociceptive mediators such as cytokines and chemokines. These gliotransmitters can sensitize neurons by activating their homologous receptors, thereby promoting central sensitivity, which is fundamental for the generation of allodynia, hyperalgesia, and spontaneous pain (Old et al., 2015). The interaction between glial cells and neurons creates an integrated network that coordinates the immune response and modulates the excitability of pain pathways (Han et al., 2015).

Recent studies have shown that acupuncture can relieve inflammatory hyperalgesia by inhibiting the interaction between spinal cord neurons and glial cells. In formalin- and CFA-induced animal models of inflammatory pain, EA can relieve pain by decreasing the expression of the pain-related factors SP, CGRP, IFN-γ, IL-6, IL-1β, and TNF-α and increasing the levels of the anti-inflammatory factors IL-4 and IL-10 in the spinal cord (Liu et al., 2019). Neuron-derived chemokine C-X3-C motif chemokine ligand 1 (CX3CL1) activates C-X3-C chemokine receptor 1 (CX3CR1) on microglia, leading to the phosphorylation of p38 MAPK in microglia (Zhang et al., 2017). EA (2 Hz, 1–2 mA) at ST36 in CFA-induced pain models can reduce pain significantly by decreasing CX3CL1 expression in the spinal cord, inhibiting the activation of the p38 MAPK pathway in microglia, and reducing the release of downstream cytokines (IL-6, IL-1β, and TNF-α) (Li et al., 2019).These cytokines are important messengers that transmit signals between glia and neurons. The reduction in IL-1β and TNF-α attenuates N-methyl-D-aspartate (NMDA) receptor phosphorylation, inhibits the change in synaptic strength, and reduces behavioral hyperalgesia (Zhang et al., 2011a; Li et al., 2019). Purinergic signaling is also involved in the maintenance of pain. After peripheral tissue injury, a significant amount of ATP is released from satellite glial cells. This ATP acts on P2 × 7R and promotes the activation of CX3CR1 in microglia, and the interaction between CX3CL1 and CX3CR1 is initiated by the activation of P2 × 7R (Clark and Malcangio, 2014). The reduction in ATP/P2 × 7R signaling observed after three session of EA inhibits the downstream CX3CL1/CX3CR1 signaling pathway and p38 MAPK phosphorylation in microglia (Gao et al., 2017). Accordingly, EA suppresses ATP/P2 × 7R/CX3CL1/CX3CR1/p38 MAPK-mediated neuroglial crosstalk and thus exerts an analgesic effect.

When pain signals are relayed to the spinal cord, glutamate released from primary neurons activates glutamate receptors on astrocytes, increasing Ca^2+^ mobilization in these cells. The release of a series of mediators from activated astrocytes in turn modulates neuronal activity. For example, IL-10 can regulate long-term potentiation (LTP) of the synapses between primary afferent C-fibers and secondary neurons in order to prevent long-term mechanical and thermal hyperalgesia (Clark et al., 2015). EA at SP6 and GB34 relieves incision-related pain and suppresses spinal LTP via an increase in IL-10 levels in spinal astrocytes (Clark et al., 2015; Dai et al., 2019). The binding of IL-33 to growth stimulation expressed gene 2 (ST2) can activate downstream MAPK signaling pathways and aggravate inflammation. This activation plays an important role in central sensitization and pain modulation (Crown, 2012). EA reduces the paw lifting time and paw licking time in mouse models of formalin-induced pain, inhibits the expression of IL-33 in astrocytes and that of ST2 in astrocytes and neurons, and further inhibits the phosphorylation of ERK and JNK. Subcutaneous or intrathecal injection of recombinant IL-33 weakens the analgesic effect of EA and reverses the EA-induced suppression of ERK and JNK phosphorylation. Therefore, EA alleviates inflammatory pain by inhibiting IL-33/ST2 signaling and the downstream ERK and JNK pathways (Han et al., 2015). Even one session of EA at ST36 and BL60 can significantly increase the pain thresholds of CFA-treated rats and remarkably suppress ERK1/2 activation and cyclo-oxygenase 2 (COX-2) expression. Three sessions of EA decrease NK-1 expression and the DNA binding activity of CREB, a transcription factor downstream to ERK1/2. Therefore, acupuncture produces an analgesic effect by preventing the activation of the ERK1/2-COX-2 pathway and ERK1/2-CREB-NK-1 pathway at different stages of inflammatory pain progression (Fang et al., 2014).

These studies together show that acupuncture inhibits the communication and interaction between glial cells and neurons by reducing the phosphorylation of p38 MAPK, inhibiting the ERK and JNK pathways, decreasing the levels of pro-inflammatory factors, and increasing the levels of anti-inflammatory factors, thereby relieving inflammatory pain.

### Regulation of Neurotransmitters in the Central Nervous System by Acupuncture

#### Glutamate

Glutamate is an excitatory neurotransmitter that is widely distributed in the CNS, and it plays a key role in the induction of CNS sensitization. Glutamate has three receptors, namely NMDA receptors, α-amino-3-hydroxy-5-methyl-4-isoxazole-propionic acid receptors, and kainate or metabotropic receptors (Swartjes et al., 2011). IL-1β and TNF-α, which are released from activated glial cells after the induction of inflammatory pain, lead to the phosphorylation of glutamate receptors. This enhances the excitability of spinal neurons and promotes pain transmission via the regulation of glutamate receptor activity and Ca^2+^-dependent signaling (Zhou et al., 2001; Liu et al., 2015). EA and acupoint catgut embedding (ACE) can inhibit the phosphorylation of the GluN1 subunit and thus inhibit the activation of NMDA receptors in the spinal cord. Further, these treatments can also inhibit Ca^2+^-dependent signals (calmodulin-dependent protein kinase II, ERK, and CREB) in CFA-treated rats, thus relieving inflammatory pain (Cui et al., 2019). In CFA-induced rat models of inflammatory pain, there is an increase in GluR2 phosphorylation in the ipsilateral SDH. EA (2 Hz, 1 mA) at ST36 and SP6 produces analgesic effects through the down-regulation of GluR2 phosphorylation (Lee et al., 2013). In addition, glutamate transporter (GT-1) in astrocytes prevents excessive activation of postsynaptic glutamate receptors by buffering the glutamate released into synapses (Nie and Weng, 2009). Nevertheless, injuries result in decreased GT-1 expression and an alteration of the glutamate homeostasis in synapses between astrocytes and neurons, leading to increased dorsal horn excitability and the development of inflammatory pain (Sung et al., 2003). In CFA-induced models of inflammatory pain, EA treatment can increase GT-1 expression in astrocytes, resulting in the clearance of excess glutamate in the synaptic cleft and a reduction in pain signals (Kim et al., 2012). Taken together, these data suggest that acupuncture modulates inflammatory pain by reducing neuroglial interactions and inhibiting the expression and phosphorylation of glutamate receptors and that it promotes glutamate reuptake by increasing the expression of glutamate transporters in astrocytes.

#### Endogenous Opioids

The activation of the endogenous opioid system is the best-understood mechanism underlying acupuncture-induced analgesia. This system mainly involves ENDs, ENKs, and DYNs, and the μ-, δ-, and κ-opioid receptors (Zhang et al., 2016). Different frequencies of EA are known to activate different opioid receptors. Low-frequency (2 Hz) EA promotes the release of ENKs, which binds to μ- and δ-opioid receptors, while high-frequency (100 Hz) EA promotes the release of DYNs, which bind to μ-opioid receptors (Seo et al., 2013; Zhang et al., 2014). Acupuncture can reduce inflammatory pain by activating different opioid receptors in different inflammatory pain models. In CFA- and capsaicin-induced inflammatory pain models, the inhibition of thermal and mechanical hyperalgesia caused by EA is blocked by the intrathecal administration of μ- and δ-opioid receptor antagonists but not by that of κ-opioid receptor antagonists (Zhang et al., 2004; Kim et al., 2009). In carrageenan-induced inflammatory pain models, the analgesic effects of acupuncture are blocked by the intrathecal administration of a μ-opioid receptor antagonist but not by that of a δ- or κ-opioid receptor antagonist (Yang et al., 2011). The differential involvement of opioid receptors might be due to changes in receptor sensitivity during the development of inflammatory pain. Interestingly, EA induces the release of ENDs in CFA-treated rats and inhibits the release of γ-aminobutyrate (GABA) by activating μ-opioid receptors on GABAergic neurons, thus activating serotonergic neurons in the rostral ventromedial medulla and causing them to release 5-hydroxytryptamine (5-HT) and suppress pain (Zhang et al., 2011b). This indicates that opioids and their receptors contribute to the increased release of analgesic transmitters, producing analgesic effects.

#### Endocannabinoids

The endocannabinoid system is involved in the control of pain transmission and is largely dependent on two ligands: N-arachidonoylethanolamide and 2-arachidonoyl glycerol (2-AG) (Finn et al., 2021). CB1R is a cannabinoid receptor that is widely distributed in the nerve endings of both GABAergic and glutamatergic neurons in the periaqueductal gray (PAG; Hu et al., 2014; Samineni et al., 2017). EA (2 Hz, 1 mA) at Ex-LE4 *(Neixiyan)* and ST35 relieves the inflammatory pain caused by KOA via an increase in the levels of 2-AG, the induction of CB1R expression on GABAergic neurons (but not on glutamatergic neurons), and a reduction in 5-HT levels. The microinjection of CB1R antagonists in the ventrolateral PAG (vlPAG) can reverse the effects of EA. In GABA-CB1^–/–^ mice, 5-HT levels do not increase after EA stimulation, confirming that EA potentiates the pain-inhibition effects of 5-HT in the descending inhibition pathway via CB1Rs on GABAergic neurons (Yuan et al., 2018b). EA can also simultaneously and bidirectionally inhibit GABAergic neurons and excite glutamatergic neurons by increasing CB1R expression in the vlPAG, thereby allowing serotonergic neurons to be sufficiently excited, resulting in antinociceptive effects (Zhu et al., 2019). Repeated EA at ST36 and BL60 increases the gene expression of *CB1R* and dopamine D1 and D2 receptors in the striatum. The injection of CB1R antagonists reverses the analgesic effects of repeated EA stimulation, indicating that the endocannabinoid system contributes to acupuncture-induced inflammatory pain reduction via the regulation of dopamine release in the striatum (Shou et al., 2013).

Therefore, endocannabinoid-CB1R-GABAergic/glutamatergic neurons and neurotransmitters (5-HT, dopamine, and norepinephrine) may form a novel pathway that mediates the acupuncture-induced inhibition of inflammatory pain. Future research should focus on the role of the endocannabinoid system in neural circuits and its interactions with other systems.

### 5-Hydroxytryptamine

Serotonergic neurons in the raphe nucleus release 5-HT, targeting receptors along the descending pain circuit, and participate in the regulation of pain perception (Boadas-Vaello et al., 2017; Tao et al., 2019). Countless studies have shown that 5-HT and 5-HT receptors (5-HTRs) participate in acupuncture-induced analgesia. EA at GB30 in CFA-treated rats can activate serotonergic neurons in the nucleus raphes magnus and cause them to release 5-HT, which binds to spinal 5-HT1ARs and produces analgesic effects. Moreover, serotonin depletion and treatment with a 5-HT1AR antagonist prevent the effects of EA (Li et al., 2007; Zhang et al., 2011c). In collagenase-induced osteoarthritis rat models, EA at ST36 produces analgesic effects, and the analgesic effect of 2-Hz EA is reduced after pretreatment with 5-HT1R and 5-HT3R antagonists, although no such effect is observed when 5-HT2R antagonists are used (Seo et al., 2016). In CFA-induced pain models, 10-Hz EA activates 5-HT1ARs, but not 5-HT2BRs, 5-HT2CRs, or 5-HT3Rs in the spinal cord (Zhang et al., 2012b). Therefore, EA inhibits hyperalgesia by activating serotonergic neurons in the spinal cord and causing them to release 5-HT, which acts on 5-HT1ARs in the spinal cord.

The activation of 5-HT1AR has been reported to block GluN1 phosphorylation (Liu et al., 2011), and EA and ACE also inhibit the activation of GluN1. Importantly, intrathecal injection of a 5-HT1AR antagonist and agonist can block and mimic, respectively, the effects of EA and ACE. EA can alleviate inflammatory pain by activating 5-HT1ARs and preventing the phosphorylation of GluN1 and Ca^2+^-dependent signaling (Cui et al., 2019). Taken together, the data suggest that 5-HT mediates the effects of acupuncture against inflammatory pain through the activation of various receptor subtypes and the inhibition of glutamate receptor phosphorylation.

### Regulation of Emotions and Cognition Associated With Pain by Acupuncture

According to the International Association for the Study of Pain, pain has sensory, emotional, cognitive, and social components. Anxiety, depression, and other negative emotions can be caused by nociceptive stimulation (Williams and Craig, 2016). The main brain region associated with pain-related emotions is the anterior cingulate cortex (ACC; Zeng et al., 2018). NMDARs and μ-opioid receptors are co-expressed in the ACC, and EA at GB30 relieves pain-induced place aversion in CFA-treated rats through the promotion of μ-opioid receptor expression and inhibition of NMDA excitation (Zhang et al., 2012a). Protein kinase M zeta (PKM zeta) and GluR1 are involved in pain and the neuroplasticity induced by pain-related emotions (Adzovic and Domenici, 2014). EA at ST36 and BL60 inhibits the phosphorylation of PKM zeta and its downstream target GluR1 and reduces ACC-mediated LTP, thereby alleviating the anxiety-like behavior induced by inflammatory pain (Du et al., 2017). The neuropeptide S/neuropeptide S receptor (NPS/NPSR) system is involved in the regulation of the anxiety induced by chronic inflammation. EA at ST36 and BL60 enhances ipsilateral NPS and NPSR protein expression in the ACC and reduces the anxiety-like behavior associated with pain (Du et al., 2020).

Pain memory is an endogenous factor in intractable chronic pain. The secondary messenger cAMP, its downstream protein kinase PKA, and the transcription factor CREB regulate pain-related learning and memory and neuroplasticity (Wu et al., 2013). EA can modulate pain memory by inhibiting the cAMP/PKA/CREB signaling pathway (Shao et al., 2016). Therefore, there has been a switch from the conventional notion of a single mode of nociceptive sensation to a multi-dimensional mode of pain–emotion–cognition in studies involving pain research. Acupuncture can not only reduce pain sensation, but can also help manage pain-related emotion and cognition by regulating neurotransmitters and neuroplasticity, thus alleviating pain and related factors in an all-round manner.

**FIGURE 4:**
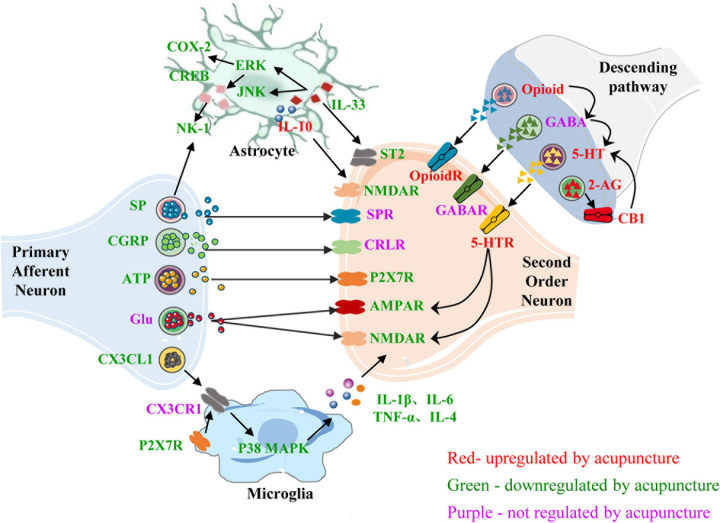
Role of neuroimmune crosstalk within the central nervous system in mediating the anti-inflammatory and analgesic effects of acupuncture on inflammatory pain. The names of neurotransmitters, neuropeptides, and immune factors are presented in red, green, and purple, respectively. The factors in red are up-regulated by acupuncture, while those in green are down-regulated by acupuncture. The factors in purple are not regulated by acupuncture. SP, substance P; SPR, substance P receptor; NK1, neurokinin-1; CGRP, calcitonin-gene-related peptide; CRLR, calcitonin-gene-related peptide receptor; ATP, adenosine triphosphate; P2 × 7R, purinergic P2 × 7 receptor; CX3CL1, C-X3-C motif chemokine ligand 1; CX3CR1, C-X3-C chemokine receptor 1; p38 MARK, p38-mitogen-activated protein kinase; COX-2, cyclo-oxygen-ase 2; CREB, cAMP response element binding protein; ERK, extracellular signal-regulated kinases; JNK, c-Jun-N-terminal kinase; AMPAR, α-amino-3-hydroxy-5-methyl-4- isoxazolepropionic acid receptor; NMDAR, N-methyl-D-aspartate receptor; GABA, γ-aminobutyrate; 5-HT, 5-hydroxytryptamine; 5-HTR, 5-hydroxytryptamine receptor; CB1, cannabinoid 1; CB1R, cannabinoid receptor 1; OpioidR, opioid receptor; GABA, γ-aminobutyrate; GABAR, γ-aminobutyrate receptor; 2-AG, 2-arachidonoyl glycerol; IL-1β/4/6/10, interleukin-1β/4/6/10; TNF-α, tumor necrosis factor-α.

In the CNS, neurons and glial cells contribute to the neuroimmune interactions involved in the production and maintenance of inflammatory pain. Acupuncture can reduce the levels of neuropeptides released by primary neurons, reduce the release of immune mediators from activated glial cells, and inhibit the interaction between glial cells and secondary neurons, thus inhibiting the transmission of pain signals. Acupuncture can also inhibit the release of central excitatory neurotransmitters and promote the release of inhibitory neurotransmitters, reduce the interaction between neurotransmitters, and relieve pain and pain-related emotions and cognition (Figure 4). Current evidence has confirmed that acupuncture regulates the glial cell–cytokine–neuron interaction. Most studies on the regulation of neurotransmitters and neuropeptides by acupuncture focus on the interactions between neurotransmitters and neural circuits. There are few studies on the relationship between neurotransmitters and glial cells, and more attention should be paid to this aspect in future studies.

## Conclusion

With in-depth research on the mechanism underlying the effects of acupuncture in treating inflammatory pain, we now fully appreciate the importance of neuroimmune interactions in this process. In peripheral regions, injury and inflammatory responses are the root causes of pain. Acupuncture can reduce the activation of pain pathways by promoting the balance between immune cells and inhibiting inflammatory responses. Acupuncture can also recruit immune cells that secrete analgesic neurotransmitters, which then act on receptors to produce analgesic effects. Long-term stimulation during peripheral inflammation leads to central sensitization. Acupuncture activates local neuroimmune regulation at acupoints and transmits acupuncture signals to the CNS. By regulating the release of neuropeptides and the interaction between glial cells and neurons, acupuncture inhibits neuroimmune crosstalk, which is vital to central sensitization. Meanwhile, acupuncture also promotes the release of analgesic neurotransmitters, inhibits the release of pain-promoting neurotransmitters, reduces the excitability of neurons and synaptic strength, and changes pain sensitivity. Accordingly, the regulation of neuroimmune crosstalk at the peripheral and central levels mediates the anti-inflammatory and analgesic effects of acupuncture on inflammatory pain in an integrated manner.

Although the reviewed studies provide reliable evidence for the application of acupuncture in the treatment of inflammatory pain, they have some limitations. First, the brain mechanisms underpinning the regulation of neuroimmune interactions by acupuncture during the treatment of inflammatory pain are not as well-studied as peripheral and spinal mechanisms. In cases of inflammatory pain, many inflammatory mediators at the inflamed site and in the spinal cord participate in the acupuncture-regulated neuroimmune crosstalk, but whether these mediators play a similar role in the brain remains unclear. Second, the interaction between spinal glial cells and neurons is involved in the acupuncture-mediated reduction in inflammatory pain, but microglia and astrocytes produce different mechanistic effects after acupuncture. It is still unclear which factors influence the timing of their activation and the molecular signal transduction within the cell. Third, some neurotransmitters can also be produced by glial cells, and glial cells also express the homologous receptors of neurotransmitters. However, it is not clear whether any interaction between neurotransmitters and glial cells mediates the effects of acupuncture in the treatment of inflammatory pain. Finally, current animal models have drawbacks because they show an acute inflammatory response and short-lived hyperalgesia, both of which become attenuated over time. Most studies have only examined the protective effect of acupuncture in the initial phase of inflammatory pain. Better models are needed to explore the effectiveness of acupuncture against chronic inflammatory pain.

Overall, this review of studies published over the last decade provides strong evidence for the usefulness of acupuncture in the treatment of inflammatory pain. The elucidation of the mechanisms underlying the effects of acupuncture in the treatment of inflammatory pain will open a variety of opportunities for further applications of acupuncture and a combination of acupuncture and drugs for treating, managing, and controlling inflammation and pain. Therefore, the continuation of research on this topic is extremely important.

## Author Contributions

BD and YLi: concept design, data collection, and manuscript writing. JM, NL, and WF: preparation of figures and the graphical abstract. LT, ZC, YGo, and ZL: data collection and analysis. ZX, SW, YX, YLiu, BC, YF, and YGu: language modification and review of the manuscript text. YGuo and XL: concept design and manuscript review. All authors contributed to the article and approved the submitted version.

## Conflict of Interest

The authors declare that the research was conducted in the absence of any commercial or financial relationships that could be construed as a potential conflict of interest.

## Publisher’s Note

All claims expressed in this article are solely those of the authors and do not necessarily represent those of their affiliated organizations, or those of the publisher, the editors and the reviewers. Any product that may be evaluated in this article, or claim that may be made by its manufacturer, is not guaranteed or endorsed by the publisher.

## Abbreviations

2-AG: 2-arachidonoyl glycerol
5-HTRs: 5-HT receptors
5-HT: 5-hydroxytryptamine
A1R: A1 receptor
ACE: acupoint catgut embedding
ATP: adenosine triphosphate
ACC: anterior cingulate cortex
CGRP: aalcitonin-gene-related peptide
CREB: cAMP response element binding protein
CB1R: cannabinoid receptor 1
CNS: central nervous system
CX3CL1: chemokine C-X 3-C motif chemokine ligand 1
CXCL10: chemokine C-X -C motif chemokine ligand 10
CXCR3: chemokine C-X -C motif receptor 3
CCR2: chemokine receptor 2
CIA: collagen-induced arthritis
CFA: complete Freund’s adjuvant
CX3CR1: C-X 3-C chemokine receptor 1
COX-2: cyclo-oxygenase 2
DRG: dorsal root ganglion
DYN: dynorphin
EA: electroacupuncture
ERK: extracellular signal-regulated kinases
GT-1: glutamate transporter
ST2: growth stimulation expressed gene 2
IBD: inflammatory bowel disease
IFN- γ: interferon- γ
IL-6: interleukin-6
JAK2: Janus kinase 2
JNK: Jun-N-terminal kinase
KOA: knee osteoarthritis
LTP: long-term potentiation
MA: manual acupuncture
MrgprC: Mas-associated G protein-coupled receptor C
ENK: enkephalin
MAPK: mitogen-activated protein kinase
MCP-1: monocyte chemoattractant protein-1
NGFs: nerve growth factors
NK-1R: neurokinin-1 receptor
NPS: neuropeptide S
NPSR: neuropeptide S receptor
NMDA: N-methyl -D-aspartate
NLRP3: NOD-like receptor family 3
NF κ B: nuclear factor kappa B
OA: osteoarthritis
PAG: periaqueductal gray
PKA: protein kinase A
PKC: protein kinase C
PKM zeta: protein kinase M zeta
P2 × 3R: purinergic P2 × 3 receptor
RA: rheumatoid arthritis
STAT3: signal transducer and activator of transcription 3
SDH: spinal dorsal horn
SP: substance P
Th1: T helper 1
Treg: T regulatory
TGF- β: transforming growth factor
TRPV1: transient receptor potential ion channel vanilloid 1
TNBS: trinitrobenzene sulfonic
TNF- α: tumor necrosis factor- α
TH: tyrosine hydroxylase
UC: ulcerative colitis
vlPAG: ventrolateral PAG
α 7nAChR: α 7 nicotinic acetylcholine receptor
β-ENDs: β-endorphins
GABA: γ-aminobutyrate .
