# Supplementary material for: Role of Neuroimmune Crosstalk in Mediating the Anti-inflammatory and Analgesic Effects of Acupuncture on Inflammatory Pain
Source: Front Neurosci. 2021 Aug 2;15:695670. doi: 10.3389/fnins.2021.695670 (PMC8366064; doi:10.3389/fnins.2021.695670)
Supplement: Supplementary file 1 [file Table_1.DOCX]

# Supplemental Table 1 Regulation mechanism of the anti-inflammatory and analgesic effects of acupuncture

| **Inflammatory pain model** | **Species** | **Intervention methods** | **Acupoints** | **Parameter of acupuncture** | **Pain measurement** | **Test site**  **Site + Mechanism** | **Refs.** | |  |
| --- | --- | --- | --- | --- | --- | --- | --- | --- | --- |
| CFA | Rat | EA | ST36, SP6 | 2 Hz, 1 mA for 30 min | thermal pain | Dorsal horn: c-fos↓, GFAP↓, GLAST↑, GL T-1↑, proteasome activity↓ | Kim. (2012) | |  |
| CFA | Rat | EA | GB30 | 10 Hz, 3 mA, twice for 20 min | thermal pain | Spinal cord: fos↓，serotonin- and catecholamine-containing neurons↑ | Li. (2007) | |  |
| CFA | Rat | EA | GB30 | 10 Hz, 3 mA for 20 min | thermal pain | Spinal cord: Use of α2a-AR, 5-HT1AR, α2b-AR, 5-HT2BR, and 5-HT3R antagonist affects acupuncture effect | Zhang. (2012) | |  |
| CIA | Rat | EA | ST36 | 2 Hz, 0.07 mA for 30 min | thermal pain | Use ofμ, κ, δ-opioid receptor antagonist andμ, κ, δ-opioid receptor agonist affects acupuncture effect | Seo. (2013) | |  |
| Carrageenan-induced pain | Rat | EA | ST36 | 2/100 Hz, 1-2mA | mechanical allodynia | ACC: cAMP↓, p-PKA↓, p-CREB↓ | Shao. (2016) | |  |
| CFA | Mice | EA | ST36 | 2 Hz, 1 mA for 5 min, 2 Hz, 1.5 mA for 5 min, and 2Hz, 2 mA for 20 min | —— | Serum: IgG↓, IgE↓;  Footpad: inflammatory cells↓, IFN-γ↓, TNF-α↓;  Spleen: lymphocytes↑, CD4^+^IFN-γ^+^T cells↓, T-bet↓, GATA-3↓, IFN-γ↓, IL-4↓ | Wang. (2017) | |  |
| UC | Mice | EA | RN4, ST36 | 2 to 100 Hz, 0.1 mA for 10 min | DAI | Colon tissues: TLR2↓, TLR4↓, Treg cells↑, Th17 cells↓, TNF-β↑, IL-10↑, IL-2↑, IL-6↓, IL-17A↓, IL-17F↓, RORγt↓, FOXP3↑ | Sun. (2017) | |  |
| CIA | Rat | EA | ST36, GB39 | 2 Hz, 6–7 mA for 15 min | arthritis score,  paw volume | Synovial tissue: VPAC1↑;  Spleen: Treg cell↑, Th17 cells↓ | Zhu. (2015) | |  |
| CFA | Rat | EA | GB30 | 30 Hz, 2 mA for 30 min | thermal pain | Inflamed paw: Use of opioid receptor, beta-endorphin and a corticotropin-releasing factor antagonist affects acupuncture effect | Zhang. (2005) | |  |
| CFA | Rat | EA | GB30, GB34 | 2/100 Hz, 1 mA for 30 min | mechanical allodynia, thermal pain | Skin tissue: anandamide↑, | Chen. (2009) | |  |
| CIA | Mice | EA | ST36, SP6 | 2 Hz, 0.07 mA for 30 min | —— | Plasma: TNF-α↓;  Ankle joints, synovial membranes: A_2A_R↓, | Li. (2015) | |  |
| IBS | Rat | EA | ST37, ST25 | 2/100 Hz, 2 mA for 20 min | AWR score | Colonic myenteric plexus: P2X_3_↓, | Weng. (2015) | |  |
| CFA | Mice | EA | ST36 | 2 Hz, 1 mA for 30 min | mechanical allodynia, thermal pain | PFC&hippocampus: pNR1↑, NR2B↑, pCaMKIIα↑Hypothalamus: pCaMKIIα↑ | | Huang. (2020) | |
| CFA | Mice | EA | ST36 | 2 Hz, 2 mA for 20 min | thermal pain | Hippocampus: CBS induced H2S↓ | | Ren. (2020) | |
| CFA | Rat | EA | ST36, BL60 | alternative 2Hz/120 Hz, 1-2mA for 30 min | —— | DRG: TNF-α↓, Cacna1e↓, Cacng5↓ | | Zhou. (2020) | |
| CFA | Rat | EA | ST36, BL60 | alternative 2Hz/100 Hz, 0.5-1.5mA for 30 min | mechanical allodynia | ACC: NPS↑, NPSR↑ | | Du. (2020) | |
| CFA | Mice | EA | ST36 | 2Hz, 1mA for 15min | mechanical allodynia, thermal pain | DRG, spinal cord and thalamus: TLR2↓, pPI3K↓, pAkt↓, pmTOR↓, pERK↓, pp38↓, pJNK↓, pCREB↓, pNFκB↓, Nav1.7↓, Nav1.8↓ | | Hsu. (2019) | |
| CFA | Mice | EA | LI4 | 2Hz, 1mA for 15min | mechanical allodynia, thermal pain | PFC, hypothalamus: TRPV1↓, pPKA↓, pPI3K↓, pPKC ε↓, pERK↓, pp38↓, pJNK↓, pAkt↓, pmTOR↓, pNF κ B↓, Nav1.7↓, Nav1.8↓;  PAG: TRPV1↑, pPKA↑, pPI3K↑, pPKC ε↑, pERK↑, pp38↑, pJNK↑, pAkt↑, pmTOR↑, pNF κ B↑, Nav1.7↑, Nav1.8↑ | | Yen. (2019) | |
| CFA | Rat | ACE | ST36, GB30 | depth 0.3 cm | mechanical allodynia, thermal pain | Spinal cord: 5-HT1AR↑, GluN1↓, CaMKII↓, ERK↓, CREB↓ | | Cui. (2019) | |
| CFA | Mice | MA | ST36 | slowly rotated every 5 min for 30 min | mechanical allodynia | DRG: TRPV1↓ | | [Jádina](https://pubmed.ncbi.nlm.nih.gov/?sort=pubdate&size=100&term=Vieira+JS&cauthor_id=29859911). (2018) | |
| CFA | Mice | MA | ST36 | 30 s of lifting-thrusting,needle retention for 30 s, 30 s of twisting, and needle retention for 30 s | paw withdrawal latency | Cerebral-spinal fluid：β-endorphin↑; Acupoint area: Mast cells degranulation↑, histamine and adenosine↑ | | Huang. (2018) | |
| CFA | Rat | EA | ST36, BL60 | 2/100 Hz EA, 1 mA for the first 15 min; 2 mA for another 15 min | thermal pain | DRG: TRPV1↓, PKCε↓;  SCDH: TRPV1↓, PKCγ↓ | | Liu. (2018) | |
| CFA | Mice | EA | ST36 | 2 Hz, 1 mA for 15 min, repeated two more times | mechanical allodynia, thermal pain | DRG: GFAP↓, S100B↓, RAGE↓, pPKCε↓, COX-2↓, pERK↓, pNFκB↓ | | Liao. (2017) | |
| CFA | Mice | EA | ST36 | 100-μs square pulses of 1 mA for 15 min at 2 Hz | mechanical allodynia, thermal pain | DRG、SC: TRPV1↓, pPKA↓, pPI3K↓, pPKC↓, GFAP↓, S100B↓, RAGE↓ | | Yang. (2017) | |
| CFA | Rat | EA | ST36, BL60 | 100 Hz, and 2 Hz alternating frequencies，1-2 mA for 30 min | mechanical allodynia | vlPAG↓, RVM↓, SCDH: p-p38MAPK-IR↓ | | Hu. (2017) | |
| CFA | Rat | EA | ST36, GB34 | 2 Hz，1.5 mA for 10 min | mechanical allodynia, thermal pain | Spinal cord: apelin↑, APJ↑ | | Wang. (2016) | |
| CFA | Rat | EA | ST36, BL60 | 2/100 Hz, 1-2 mA for 30 min | mechanical allodynia | Spinal: GRK2↑ | | Liu. (2015) | |
| CFA | Rat | EA | ST36, BL60 | 2/100 Hz, 1-2 mA for 30 min | mechanical allodynia | SCDH: ERK1/2↓, COX-2↓, NK-1↓, CREB↓ | | Fang. (2014) | |
| CFA | Mice | MA | ST36 | two twists every 5 min for 30 min | mechanical allodynia, thermal pain | Acupoint: TRPV1↑, TRPV4↑, ASIC3↑ | | Wu. (2014) | |
| CFA | Rat | EA | GB30 | 100 Hz, 2–3 mA for 20 min | mechanical allodynia, thermal pain | Inflamed paw: Use of opioid receptor/peptide antagonists affects acupuncture effect | | Wang. (2013) | |
| CFA | Rat | EA | ST36, BL60 | 2 Hz/100 Hz, 1-2mA for 30 min | mechanical allodynia | Spinal dorsal horn：p-p38 MAPK↓、p-ATF-2↓、VR-1↓ | | Fang. (2013) | |
| CFA | Rat | EA | ST36, BL60 | 2 Hz/100 Hz,1-3 mA for 20 min | thermal pain | Brain Striatum: Cannabinoid CB1 Receptor Protein↑ | | Shou. (2013) | |
| CFA | Mice | EA | ST36 | 2 Hz, 1 mA for 120 min | mechanical allodynia, thermal pain | DRG Neurons: Nav1.8 channels↓, Nav1.7 channels↓ | | Huang. (2013) | |
| CFA | Rat | MA | ST36 | lift-thrusting and twisting manipulation for 30 sec each, for 30 min | thermal pain | Acupoints: Use of histamine and H1 receptor antagonist affects acupuncture effect | | Huang. (2012) | |
| CFA | Mice | EA | ST36 | 2 Hz,2 mA for 15 min | thermal pain | DRG neurons: TRPV1↓, TRPV4↓ | | Chen. (2012) | |
| CFA | Rat | EA | GB30, GB34 | 2 Hz,1 mA for 30 min | mechanical allodynia, thermal pain | Inflamed skin tissues：IL-1β↓, IL-6↓, TNF-α↓ | | Su. (2012) | |
| CFA | Rat | EA | GB30, GB34 | 2 Hz,1 mA for 30 min | mechanical allodynia, thermal pain | Inflamed skin tissues: β-endorphin↑, POMC↑, the percentage of β-endorphin-immunoreactive keratinocytes↑, macrophages↑, and T-lymphocytes↑ | | Su. (2011) | |
| CFA | Rat | EA | EX-B2 | 2 Hz/100 Hz, 1mA for 30 min | thermal pain | Hypothalamic paraventricular nucleus: CRH↑ | | Qiao. (2012) | |
| CFA | Rat | EA | ST36, BL60 | 2 Hz/100 Hz, 1-2mA for 30 min | mechanical allodynia | Ipsilateral spinal dorsal horn: p-p38MAPK-IR↓ | | Liang. (2012) | |
| CFA | Rat | EA | GB30 | 10 Hz, 3 mA for 20 min | CPA test | Spinal cord: Use of 5-HT1AR and 5-HT2CR antagonist affects acupuncture effect | | Zhang. (2011) | |
| CFA | Rat | MA | ST36, SP6 | 2 Hz, 1 mA for 20 min | mechanical allodynia, thermal pain | Spinal cords:NR2B↓, p38↓, ERK↓, CREB↓ | | Jang. (2011) | |
| CFA | Rat | EA | GB30, GB34 | 60/4 Hz, 1mA for 30 min | thermal pain | Spinal cords: NT-3↑, GFAP↓, OX-42↓, IL-1β↓, IL-6↓, TNF-α↓ | | Mi. (2011) | |
| CFA | Rat | EA | GB30, GB34 | 2/100Hz, 1 mA for 30 min | —— | Inflamed skin tissues: CB2Rs↑, | | Zhang. (2010) | |
| Formalin-induced pain | Rat | EA | ST36, BL40 | 2Hz, 1mA for 30min | mechanical allodynia  Licking time | Spinal cord: microglia↓, neurons↓, IL-6↓, IFN-γ↓, IL-4↑, SP↓, CGRP↓ | | Liu. (2019) | |
| Formalin-induced pain | Mice | EA | ST36, GB34 | 2/100 Hz, 1–2 mA for 30 min | formalin test | lumbar spinal：IL-33、ST2↓ | | HHan. (2015) | |
| Formalin-induced pain | Rat | EA | BL60 | 1 Hz,3 mA for 10 min | flinching frequency of formalin-injected paws | Spinal cord：c-Fos positive neurons↓ | | Chang. (2012) | |
| Carrageenan-induced pain | Mice | MA | SP6 | 2–3 mm, rotated slowly for 10-min | mechanical allodynia, thermal pain | Muscle: IL-10↑, M1 macrophages↓, M2 macrophages↑ | | Silva. (2015) | |
| Carrageenan-induced pain | Rat | AA | A4 | 4Hz, 0.7-1 mA for 45 min | mechanical allodynia, thermal pain | Use of opioid receptor, peripheral cholinergic muscarinic receptors antagonist affects acupuncture effect | | Chung. (2011) | |
| Carrageenan-induced pain | Rat | EA | ST36 | 3Hz,1-2-3 mA for 60 min | mechanical allodynia | Peripheral: μ, δ and κ receptors are activated | | Taguchi. (2010) | |
| Carrageenan-induced pain | Rat | EA | ST36, SP6 | 10 Hz, 1 mA for 30 min | Weight-bearing behavioral tests | Spinal cords: Use of μ, κ, δ-opioid receptor antagonist affects acupuncture effect | | Yang. (2011) | |
| MIA | Mice | EA | Ex-LE4, ST35 | 2Hz, 1mA for 30min | mechanical allodynia, thermal pain | Menisci: CB2↑, IL-1β↓ | | Yuan. (2018) | |
| MIA | Rat | EA | GB30, ST36 | 10Hz, 2 mA for 30 min | Weight-bearing behavioral tests | Spinal Cord: Serotonin-Containing NRM Neurons↑ | | Li. (2011) | |
| CIA | Rat | EA | ST36 | 2 Hz and 100 Hz, 0.07 mA for 30 min | mechanical allodynia, thermal pain | Use of 5-HT1, 5-HT2, 5-HT3, muscarinic cholinergic receptor agonist and 5-HT1, 5-HT2, 5-HT3, muscarinic cholinergic receptor antagonist affects acupuncture effect | | Seo. (2016) | |
| CIA | Rat | EA | ST36 | 2 Hz, 0.07 mA for 30min | thermal pain | Use of α2-adrenoceptor，α2-adrenoceptor and β-adrenoceptor antagonist affects acupuncture effect | | Park. (2013) | |
| Monosodium urate | Rat | EA | ST36, BL60 | 0.2 ms, 1-2 mA for 30 min | ongoing pain score evaluation, mechanical allodynia, thermal pain | local ankle skin tissue：β-endorphin↑ | | Chai. (2018) | |
| Spontaneous aging-related OA | Hartley guinea pigs | EA | Ex-LE4, ST35 | 2Hz, for 30 mins | mechanical allodynia | Cartilage tissue: NLRP3↓, Caspase1↓, IL-1β↓, MMP 13↓；  Serum: IL-1β↓, TNF-α↓ | | Wang. (2020) | |
| Spontaneous aging-related OA | New Zealand White rabbits | EA | BL18, BL20, BL23, ST36, SP6 | 10 Hz, 1-5 mA for 30 min | modified mankin score | Distal femur：MMP-13↓ | | Qin. (2015) | |
| incision pain model | Rat | MA | ST35, ST36 | twisted for 1 min, 2Hz, left 4 min for 30min | mechanical allodynia, thermal pain | Knee joint: NGF↓;  Synovia and cartilage: MCP1↓, CCR2↓, IL-1β↓, TNF-α↓ | | Li. (2020) | |
| incision pain model | Mice | EA | SP6, GB34 | 2Hz/100 Hz, 1-3mA for 30 min | mechanical allodynia | Spinal cord: IL-10↑, LTP↓ | | Dai. (2019) | |
| colitis model | Rat | EA | ST36, ST37 | 2Hz, 1mA for 30min | mechanical allodynia, thermal pain | DRG: TH↓, CGRP↓ | | Wang. (2019) | |
| colitis model | Rat | EA | ST36, ST37 | 2Hz, 1mA for 30min | mechanical allodynia, thermal pain | Spinal cord: LTP↓ | | Lv. (2019) | |
| Third lumbar vertebrae transverse process syndrome model | Rat | TENS | BL23, ST36 | 2/100 Hz，dense-disperse wave for 30 min | thermal pain | Local muscle tissues: IL-1β↓, TNF-α↓, iNOS↓ | | Li. (2015) | |
